# Neuromeric Distribution of Nicotinamide Adenine Dinucleotide Phosphate-Diaphorase Activity in the Adult Lamprey Brain

**DOI:** 10.3389/fnana.2022.826087

**Published:** 2022-02-07

**Authors:** Manuel A. Pombal, Manuel Megías, Daniel Lozano, Jesús M. López

**Affiliations:** ^1^Neurolam Group, Facultade de Bioloxía-IBIV, Departamento de Bioloxía Funcional e Ciencias da Saúde, Universidade de Vigo, Vigo, Spain; ^2^Department of Cellular Biology, Faculty of Biology, Complutense University of Madrid, Madrid, Spain

**Keywords:** *Lampetra fluviatilis*, cyclostomes, histochemistry, nitric oxide, segmentation, evolution

## Abstract

This study reports for the first time the distribution and morphological characterization of nicotinamide adenine dinucleotide phosphate-diaphorase (NADPH-d; a reliable marker of nitric oxide synthase activity) positive elements in the central nervous system of the adult river lamprey (*Lampetra fluviatilis*) on the framework of the neuromeric model and compares their cytoarchitectonic organization with that of gnathostomes. Both NADPH-d exhibiting cells and fibers were observed in all major divisions of the lamprey brain as well as in the spinal cord. In the secondary prosencephalon, NADPH-d positive cells were observed in the mitral cell layer of the olfactory bulb, evaginated pallium, amygdala, dorsal striatum, septum, lateral preoptic nucleus, caudal paraventricular area, posterior entopeduncular nucleus, nucleus of the stria medullaris, hypothalamic periventricular organ and mamillary region *sensu lato*. In the lamprey diencephalon, NADPH-d labeled cells were observed in several nuclei of the prethalamus, epithalamus, pretectum, and the basal plate. Especially remarkable was the staining observed in the right habenula and several pretectal nuclei. NADPH-d positive cells were also observed in the following mesencephalic areas: optic tectum (two populations), torus semicircularis, nucleus M5 of Schöber, and a ventral tegmental periventricular nucleus. Five different cell populations were observed in the isthmic region, whereas the large sensory dorsal cells, some cells located in the interpeduncular nucleus, the motor nuclei of most cranial nerves, the solitary tract nucleus, some cells of the reticular nuclei, and small cerebrospinal fluid-contacting (CSF-c) cells were the most evident stained cells of the rhombencephalon proper. Finally, several NADPH-d positive cells were observed in the rostral part of the spinal cord, including the large sensory dorsal cells, numerous CSF-c cells, and some dorsal and lateral interneurons. NADPH-d positive fibers were observed in the olfactory pathways (primary olfactory fibers and stria medullaris), the fasciculus retroflexus, and the dorsal column tract. Our results on the distribution of NADPH-d positive elements in the brain of the adult lamprey *L. fluviatilis* are significantly different from those previously reported in larval lampreys and demonstrated that these animals possess a complex nitrergic system readily comparable to those of other vertebrates, although important specific differences also exist.

## Introduction

Reduced nicotinamide adenine dinucleotide phosphate-diaphorase (NADPH-d) histochemistry has been extensively used for detection of specific cell populations in both the central (CNS) and peripheral nervous systems (see Alonso et al., 2000). In addition, the histochemical staining for NADPH-d was identified to be associated with nitric oxide synthase (NOS; Bredt et al., 1991a; Dawson et al., 1991; Hope et al., 1991). NOS is the enzyme that catalyzes the synthesis of nitric oxide, a gaseous messenger molecule (Bredt and Snyder, 1992), from L-arginine. There are different types of NOS: neuronal NOS (nNOS) and endothelial NOS (eNOS), which are constitutively expressed in the nervous system, and induced NOS (iNOS) in the macrophages. In the CNS of anamniotes (excluding amphibians), NO is mainly produced by nNOS, whereas eNOS, together with nNOS, contributes to produce NO in amphibians and amniotes (reviewed in Toda and Ayajiki, 2006).

The presence of NADPH-d/nNOS has been shown in many different types of neurons along the nervous system of vertebrates colocalizing with several classical neurotransmitters and neuroactive substances like acetylcholine (Vincent et al., 1983a,b, 1986; Schober et al., 1989, 1993; Villani et al., 1994), GABA (Vaney and Young, 1988; Dawson et al., 1991; Valtschanoff et al., 1993; Weiler and Kewitz, 1993) or some neuroactive peptides (Schober et al., 1994b), somatostatin (Vincent and Johansson, 1983; Vincent et al., 1983a; Kowall and Beal, 1988), neuropeptide Y (NPY; Scott et al., 1987), and galanin (Pasqualotto and Vincent, 1991). Therefore, NO can modulate not only the cholinergic and GABA-ergic synaptic transmission (Yang and Cox, 2007; Maggesissi et al., 2009; Tarasenko et al., 2014; Gasulla and Calvo, 2015; Yamamoto et al., 2015) but also the glutamatergic (Garthwaite, 1991; Lawrence and Jarrott, 1993; Rudkouskaya et al., 2010; Neitz et al., 2011; Raju et al., 2015) and dopaminergic (Zhu and Luo, 1992; Bugnon et al., 1994; Chaparro-Huerta et al., 1997; Kiss et al., 2004) neurotransmission.

In the literature, there are numerous mapping studies on the distribution of NADPH-d/nNOS positive neurons in the CNS of many vertebrate species (see Alonso et al., 2000), demonstrating wide and complex nitrergic systems where NO could exert different sensory and motor modulations. In addition, NO plays a relevant role in synaptic plasticity, like long-term potentiation and long-term depression, in the regulation of the sleep-wake cycle, and it is also related to processes of neuroprotection and neurodegeneration (Shibuki and Okada, 1991; Haley et al., 1992; Mizutani et al., 1993; McCann, 1997; Calabresi et al., 1999; Hars, 1999; Bon and Garthwaite, 2003; Stanton et al., 2003; Wang et al., 2005; Sultana et al., 2006; Mancuso et al., 2007; Sergeeva et al., 2007; Rudkouskaya et al., 2010; Hardingham et al., 2013). In general, there is a conserved pattern of NADPH-d/nNOS positive neurons distribution across vertebrates, with main populations located at pallial/cortical areas, basal ganglia, amygdaloid complex, preoptic area, hypothalamus, optic tectum/superior colliculus region, reticular formation and spinal cord. Among fishes, many studies have described the organization of the nitrergic system in teleosts (Holmqvist et al., 1994, 2000; Arévalo et al., 1995; Brüning et al., 1995; Villani and Guarnieri, 1995a,b; Anken and Rahmann, 1996; Bell et al., 1997; Virgili et al., 2001; Singru et al., 2003; Giraldez-Perez et al., 2008, 2013) and more recently for other basal actinopterygian groups (Cladistians: López et al., 2016; Holosteans: López et al., 2017) and the Australian lungfish (Sarcopterygii: Dipnoi; López et al., 2019). However, there is little documentation regarding the presence of this type of neurons in cyclostomes. In lampreys, only the distribution of NADPH-d labeled structures in the brain of larval specimens of *Lampetra planeri* (*L*. *planeri*) was described (Schober et al., 1994a). In addition, NOS activity was also reported in the olfactory mucosa of the sea lamprey, *Petromyzon marinus* (*P. marinus*; Zielinski et al., 1996). Interestingly, the NADPH-d staining obtained in the former study on developing lampreys appears rather simple and is significantly different from that reported in other vertebrates, pointing to problems with the set-up of the histochemical technique. The available results in developing lampreys raised the question on whether the organization of NADPH-d/NOS systems in these animals is very different to that reported for other vertebrates or these systems develop later (during metamorphosis) to finally get a more gnathostome-like pattern. To solve this question, we carried out a detailed study on the distribution of NADPH-d positive elements in the brain of an adult lamprey species, the river lamprey *L. fluviatilis*, by using the histochemical method. In addition, the common and distinct features of the nitrergic system in lampreys, as compared with those of other vertebrates, are discussed.

## Materials and Methods

### Nicotinamide Adenine Dinucleotide Phosphate-Diaphorase Histochemistry

This study was performed on adult river lampreys, *Lampetra fluviatilis* (*L. fluviatilis*; *n* = 8) of both sexes. The original research reported herein was performed according to the regulations and laws established by European Union (2010/63/EU) and Spain (Royal Decree 118/2021) for care and handling of animals in research. Prior to experiments, the animals used were anesthetized with 0.01% tricaine methanesulfonate (MS-222, Sandoz, Basel, Switzerland; pH 7.3). The brain and the upper spinal cord were quickly removed and fixed by immersion in 4% paraformaldehyde in 0.1 M phosphate buffer (PB, pH 7.4) for 2–4 h at room temperature. After fixation, the brains were washed in 0.1 M PB, pH 7.4, and immersed in a solution of 30% sucrose in PB for 4–6 h at 4°C, until they sank. They were then embedded in a solution of 15% gelatin with 30% sucrose in 0.1 M PB, pH 7.4, and stored for 5 h in a 4% formaldehyde solution in the same buffer at 4°C. The gelatin blocks were cut on a freezing microtome at 40 μm in the transverse or sagittal plane and sections were collected in 0.1 M PB, pH 7.4. Free-floating sections were rinsed in fresh PB and incubated in a medium made up of 1 mM β-NADPH (Merck; Sigma-Aldrich, Poole, United Kingdom), 0.8 mM nitro blue tetrazolium (Merck; Sigma-Aldrich, Poole, United Kingdom) and 0.06% Triton X-100 in PB, at 37°C in darkness for 1–2 h. The reaction was stopped by successive rinses in cold PB. Some sections were incubated in a medium without β-NADPH. A second group of control sections was heated in PB to 70°C for 10 min. In both cases, no reaction was observed. All sections were then mounted on slides (mounting medium: 0.25% gelatin in 0.1 M Tris buffer, pH 7.6), dried overnight and coverslipped with Entellan (Merck, Darmstadt, Germany).

### Evaluation and Presentation of the Results

**FIGURE 1:**
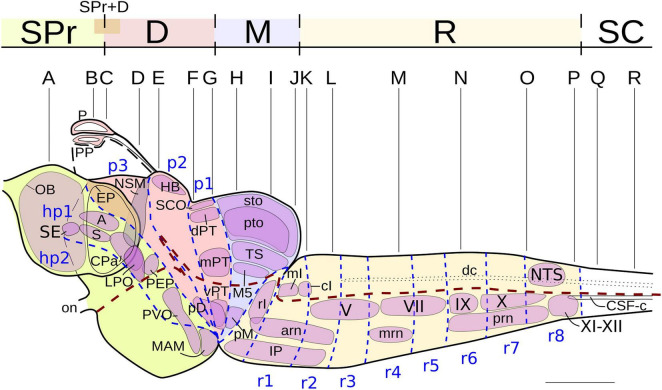
Lateral view of the brain and rostralmost spinal cord of the lamprey *Lampetra fluviatilis*, showing the NADPH-d labeled nuclei (thin continuous black lines). The dashed blue lines mark putative neuromeric subdivisions (modified from Pombal et al., 2001, 2009; Murakami et al., 2004) and the thick dashed brown line represents the alar-basal boundary. The levels of the transverse sections shown in Figure 2 are also indicated. Rostral direction is oriented to the left. For abbreviations, see list. Scale bar = 1 mm.

**FIGURE 2:**
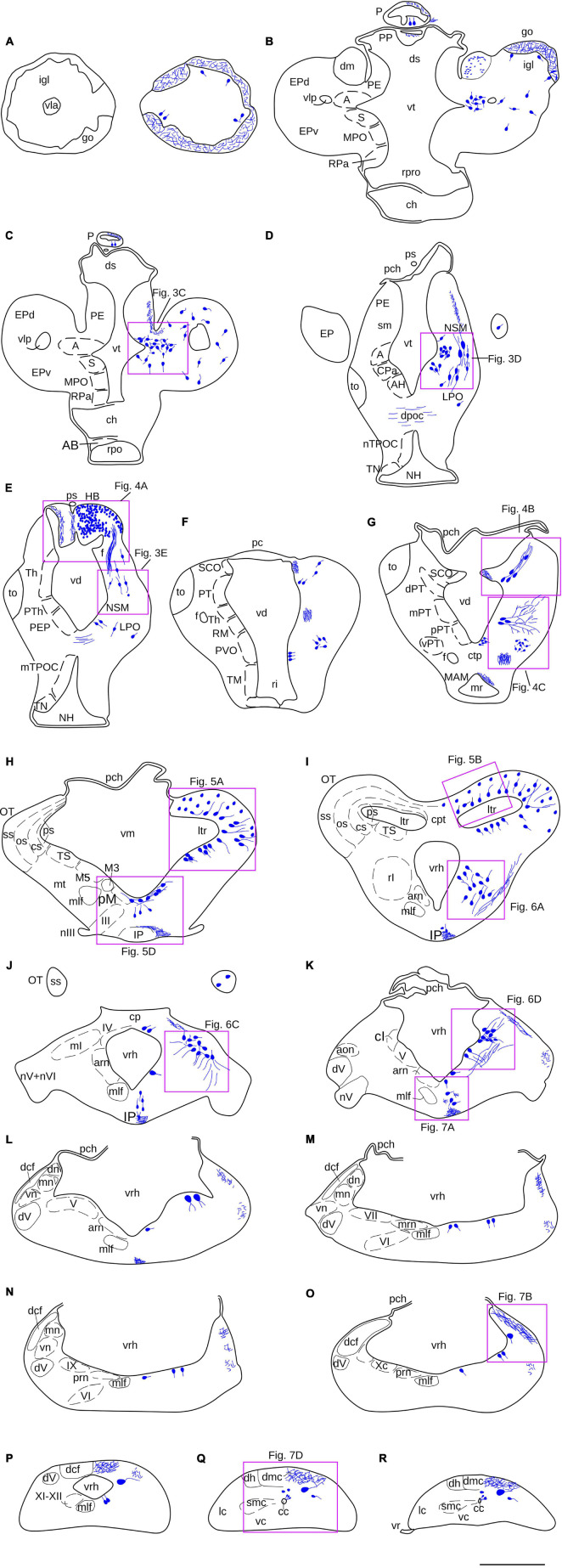
**(A–R)** Schematic drawings of a series of transverse sections through the brain and rostralmost spinal cord of *L. fluviatilis*, at the rostrocaudal levels indicated in Figure 1. The distribution of NADPH-d positive cell bodies (large blue dots) and fibers (small blue dots, wavy blue lines) are represented in the right half of each section, whereas some cell populations and specific regions or fiber tracts are represented in the left half. The relative location of the transverse illustrations shown in Figures 3–7 are indicated by purple rectangles. For abbreviations, see list. Scale bar = 1 mm.

Additional series of lamprey brains from our collection stained with cresyl violet or hematoxylin-eosin were available for topographical purposes. All the sections were examined with a BX51 Olympus microscope (Olympus; Tokyo, Japan). Photomicrographs were taken with a DP-11 Olympus digital camera. The images were subsequently adjusted for brightness and contrast with GIMP 2.10^1^. The final photomontage and lettering were done with Inkscape^2^. The distribution of NADPH-d positive cell bodies and fibers is illustrated in drawings of a sagittal view of the brain (Figure 1) and representative transverse sections (Figure 2) at the levels indicated in Figure 1. The cell sizes reported herein were measured by using a calibrated eye piece. In general, we subdivided the labeled neurons into three different size categories: small (≤10 μm), medium (between 10 and 25 μm), and large (≥25 μm). The term giant is also used for the Müller and Mauthner cells. The nomenclature of Pombal and Puelles (1999), with the modifications introduced by Pombal et al. (2009) and Martínez-de-la-Torre et al. (2011) is followed for the forebrain, whereas that used by Pombal et al. (1997, 2001) is utilized for the brainstem and spinal cord.

## Results

Most of the NADPH-d labeled cell populations detected in the lamprey CNS were located in the forebrain and midbrain, but some NADPH-d positive cells were also identified in the hindbrain (particularly in the isthmic region), and in the rostral spinal cord. Stained fibers were present in tracts in different regions of the brain. Variable NADPH-d staining intensities were observed throughout the brain and rostral spinal cord, ranging from very faint to Golgi-like staining. Even when the reaction time during the histochemical procedure was long, differences in intensity between cell groups were maintained. In general, long reaction times favored the visualization of weakly reactive cells and fibers but some background staining was also added. The specificity of the staining in distinct cell populations allowed us to better describe some poorly differentiated areas of the lamprey CNS.

**FIGURE 3:**
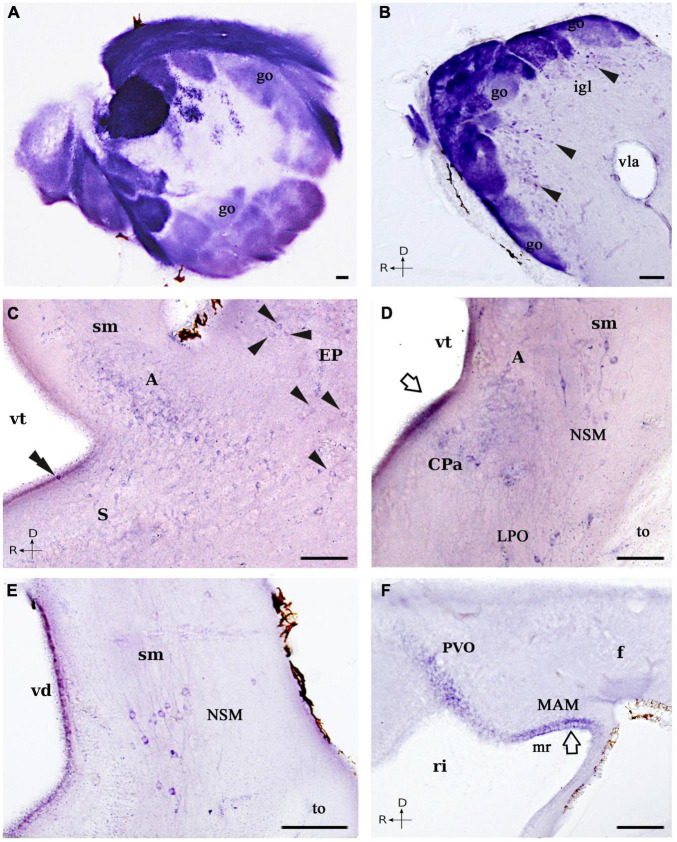
Photomicrographs showing NADPH-d positive cells and fibers in the forebrain of *L. fluviatilis*. **(A,B)** Transverse **(A)** and sagittal **(B)** sections showing a strong NADPH-d staining in most of the primary olfactory fibers and the olfactory glomeruli. Note that particular glomeruli show different intensities of reaction, as well as the presence of positive mitral cells (arrowheads) bordering the glomeruli in **(B)**. **(C)** Transverse section showing NADPH-d positive cells in the amygdala (A), the dorsal striatum (S), and the evaginated pallium (EP; arrowheads). A positive CSF-c cell is present in the striatum (double arrowhead). **(D)** Transverse section showing NADPH-d positive cells in the caudal amygdala (A), the nucleus of the stria medullaris (NSM), the caudal paraventricular area (CPa), and the lateral preoptic nucleus (LPO). The open arrow points to the periventricular staining. **(E)** Transverse section illustrating part of the cells included in the nucleus of the stria medullaris (NSM). **(F)** Sagittal section showing weak NADPH-d labeled cells in the hypothalamic periventricular organ (PVO) and in the mamillary region *sensu lato* (MAM), as well as positive fibers in the fasciculus retroflexus (f). Medial and rostral directions are oriented to the left in transverse and sagittal sections, respectively. For abbreviations, see list. Scale bar = 50 μm.

**FIGURE 4:**
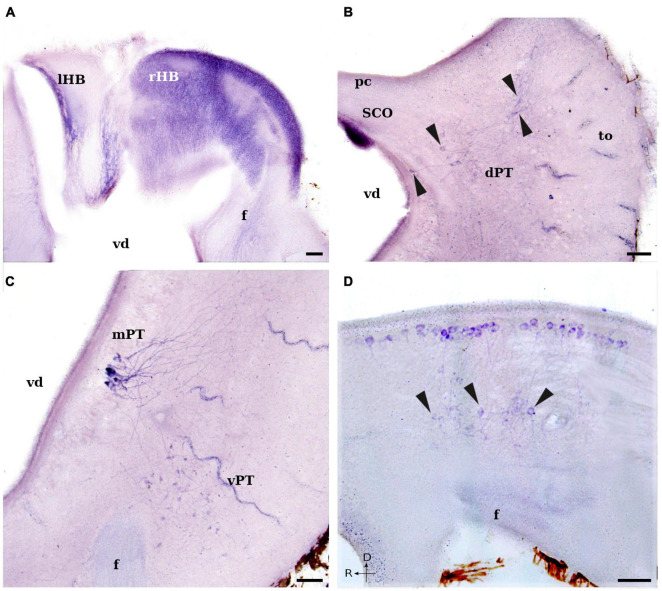
Photomicrographs showing NADPH-d positive cells and fibers in the diencephalon of *L. fluviatilis*. **(A)** Transverse section through the habenula showing numerous and small NADPH-d positive cells in the right part (rHB; the largest part), whereas only some positive fibers are present in the left part (lHB). Some positive fibers are also observable in the fasciculus retroflexus (f). **(B)** Transverse section showing a strong NADPH-d staining in the subcommissural organ (SCO), as well as some positive cells (arrowheads) in the dorsal pretectal nucleus (dPT). Note the area of distribution of the labeled cells and their dendrites. **(C)** Transverse section showing strong NADPH-d positive cells in the medial pretectal nucleus (mPT) and more weakly stained cells in the ventrolateral pretectal nucleus (vPT). Note also the presence of weakly labeled fibers in the fasciculus retroflexus (f). **(D)** Sagittal section showing several NADPH-d labeled periventricular cells covering the rostrocaudal extension of the ventral diencephalon. Some ventral displaced cells (arrowheads) are located dorsally to the fasciculus retroflexus (f). Medial and rostral directions are oriented to the left in transverse and sagittal sections, respectively. For abbreviations, see list. Scale bar = 25 μm.

**FIGURE 5:**
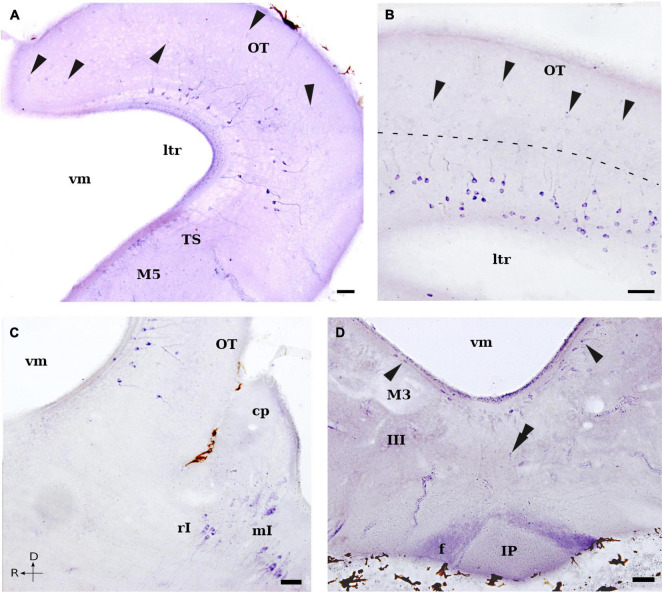
Photomicrographs showing NADPH-d positive cells and fibers in the mesencephalon and isthmic region of *L. fluviatilis*. **(A)** Transverse section showing strong NADPH-d positive cells in the deeper layers of the optic tectum (OT) and weaker stained cells (arrowheads) in the superficial layers. Note also the presence of several subependymal cells in the torus semicircularis (TS), as well as a few in the nucleus M5 of Schöber (M5). **(B)** Transverse section through the caudalmost optic tectum (OT) showing the morphology and distribution of the two types of cells labeled in this structure, separated by a dashed line (arrowheads point to weaker stained superficial cells). **(C)** Sagittal section showing NADPH-d labeled cells in the caudal optic tectum (OT), as well as in the isthmic region: rostral isthmic nucleus (rI) and medial isthmic nucleus (mI). **(D)** Transverse section showing NADPH-d positive periventricular cells in the ventral mesencephalic tegmentum (arrowheads). Note the presence of a ventral displaced labeled cell (double arrowhead). Abundant labeled fibers of the fasciculus retroflexus (f) enter the rostral part of the interpeduncular nucleus (IP). Medial and rostral directions are oriented to the left in transverse and sagittal sections, respectively. For abbreviations, see list. Scale bar = 25 μm.

**FIGURE 6:**
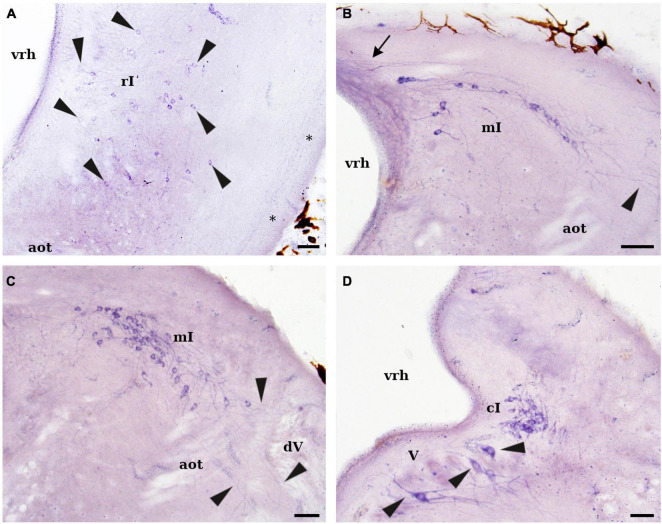
Photomicrographs showing NADPH-d positive cells and fibers in the isthmic region of *L. fluviatilis*. **(A)** Transverse section showing NADPH-d labeled cells (arrowheads) in the rostral isthmic region (rI). These cells distribute in a wide area of the isthmic tegmentum. Note also a labeled tract coursing in the lateral margin of the tegmentum (asterisks). **(B)** Transverse section showing NADPH-d positive cells in the medial isthmic region (mI). Processes of these cells are seen coursing to the contralateral side (through the so-called cerebellar commissure; arrow) or ramifying in the lateral part of the brain (arrowhead). **(C)** Transverse section of the caudal part of the same nucleus (mI) showing lateral dendrites ramifying close to the entrance of the trigeminal nerve roots (arrowheads). Thick unlabeled axons of the anterior octavomotor tract (aot) can be also identified. **(D)** Transverse section showing NADPH-d positive cells of the caudal isthmic nucleus (cI) located at pretrigeminal level. Note also the presence of some clearly positive cells in the trigeminal motor nucleus (V; arrowheads). Medial direction is oriented to the left in all transverse sections. For abbreviations, see list. Scale bar = 25 μm.

**FIGURE 7:**
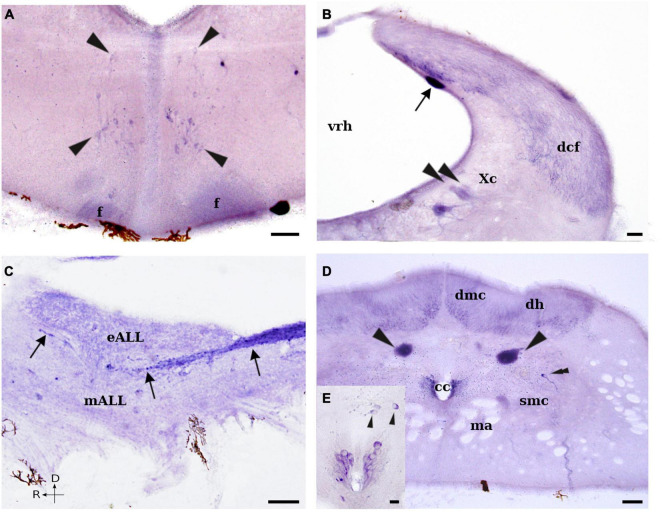
Photomicrographs showing NADPH-d positive cells and fibers in the rhombencephalon and rostral spinal cord of *L. fluviatilis*. **(A)** Transverse section showing some NADPH-d positive cells (arrowheads) close to the midline in the rostral part of the rhombencephalon. Note also the presence of labeled fibers in the fasciculus retroflexus (f). **(B)** Transverse section through the caudalmost rhombencephalon showing a strong labeled sensory dorsal cell (arrow) and many labeled fibers in the dorsal column tract (dcf). Note also the presence of some weakly labeled cells in the caudal part of the vagal motor nucleus (Xc; arrowheads). **(C)** Sagittal section showing NADPH-d labeled fibers of the dorsal column tract ascending toward the dorsal isthmic region (arrows). **(D)** Transverse section showing NADPH-d positive cells in the rostral part of the lamprey spinal cord. Two strong NADPH-d labeled sensory dorsal cells are located in each side of the cord (arrowheads), whereas a single labeled cell (double arrowhead) is present dorsolateral to the spinal motor column. Note also the presence of several labeled CSF-c cells at both sides of the central canal (cc), as well as many labeled fibers in the dorsomedial column (dmc) and the dorsal horns (dh). **(E)** Higher magnification of the central canal of a parallel section showing the labeled CSF-c cells as well as some non-CSF-c cells dorsally displaced (arrowheads). Medial and rostral directions are oriented to the left in transverse and sagittal sections, respectively. For abbreviations, see list. Scale bar = 25 μm.

A lateral view of the brain and rostral spinal cord of *L. fluviatilis* is shown in Figure 1, whereas the NADPH-d labeled perikarya and fibers are represented schematically in Figure 2 by using selected transverse sections at the levels shown in Figure 1. The areas framed in Figure 2 are shown in Figures 3–7 revealing the more remarkable NADPH-d labeling in the lamprey CNS.

### Secondary Prosencephalon

#### Perikarya

In the olfactory bulbs, numerous NADPH-d positive cells were observed surrounding the olfactory glomeruli. These cells were medium-sized (10–25 μm), round, fusiform, or polygonal and located internally to the glomeruli (Figures 1, 2A,B, 3A,B); thus, these cells appear to correspond to mitral cells, the main efferent component of the olfactory bulbs. In the telencephalon proper, numerous and scattered faintly labeled individual cells appeared all through the evaginated pallium, covering both the dorsal and the ventral portions (EP; Figures 1, 2B–D, 3C). They were round, small (10 μm in diameter), and more abundant in the caudal part of the evaginated pallium.

The most conspicuous telencephalic population of NADPH-d positive cells was found in the amygdala (A; Figures 1, 2B–D, 3C,D; see Martínez-de-la-Torre et al., 2011). They were small (10 μm in diameter), round shaped and weakly labeled, with some of them located very close to the ventricular surface, just beneath the ependymal cells. A few of them were multipolar, but in most cases, only the proximal part of a main process could be distinguished. In sagittal sections, this population was observed reaching the ventromedial aspect of the olfactory bulb, where some positive cells lay close to the anterior lateral telencephalic ventricle. Immediately ventral to this region, some labeled cells of the same size distributed in the most dorsal and dorsolateral parts of the striatal region (S; Figures 2B,C, 3C). A few of these cells appear to be cerebrospinal fluid-contacting (CSF-c) cells, with their somata located between the ependymal cells.

Rostral to the striatum, some NADPH-d labeled cells were located at both sides of the ventral midline, in the region that may represent the septum (SE; Figure 1) of the lamprey brain. These cells were small (8–10 μm) and appeared to be of CSF-c type. Dorsal to the caudal part of the postoptic commissure, there were a few medium to large (20–25 μm), bipolar NADPH-d labeled cells that we have identified as belonging to the entopeduncular nucleus. Although they were weakly stained, two main processes could be distinguished, one directed dorsally and another coursing into the postoptic commissure (PEP; Figures 1, 2E). At more dorsal levels, small (10 μm in diameter) NADPH-d positive cells were present in the caudal paraventricular area, and some of them could also be CSF-c cells due to the presence of a diffuse staining in the ventricular surface (CPa; Figures 1, 2D, 3D; see Pombal et al., 2001, 2009).

Ventrolaterally, some medium-sized (15–20 μm) and scattered NADPH-d positive cells were detected medial to the optic tract. They were mainly spindle-shaped with the main axis directed dorsoventrally, and were located midway between the ventricle and the external surface of the brain (LPO; Figures 1, 2D,E, 3D). We have tentatively identified these cells as part of the lateral preoptic nucleus. A long band of medium-sized (20 μm) and migrated NADPH-d positive cells was seen from lateral to the caudal amygdala to the proximity of the habenula. They were also bipolar and intermingled with labeled fibers of the stria medullaris, into which their processes appear to be incorporated (NSM; Figures 1, 2D,E, 3D,E; see below). Due to their position, we identified these cells as belonging to the nucleus of the stria medullaris (Pombal and Puelles, 1999).

The NADPH-d staining was also found in the basal hypothalamus of the river lamprey. Some weakly labeled cells were observed close to the ventricle in the hypothalamic periventricular organ (PVO; Figures 1, 2F, 3F; Pombal and Puelles, 1999). Although no apical dendrites directed to the ventricular surface were clearly distinguished, these cells appear to be of CSF-c type. In addition, numerous small (around 8 μm in diameter) CSF-c cells were present in the mamillary region *sensu lato* (MAM; Figures 1, 2G, 3F; Pombal and Puelles, 1999). They were weakly stained and located in the dorsal part of the mamillary recess. These two hypothalamic cell populations were better distinguished in sagittal sections (Figure 3F).

#### Fibers

In the adult lamprey olfactory bulb, almost all primary olfactory fibers and glomeruli were strikingly dark-labeled for NADPH-d (Figures 2A,B, 3A,B). However, the NADPH-d activity was heterogeneous in the different afferent fiber bundles, with the strongest labeled being located medially (Figure 3A). The so-called dorsomedial telencephalic neuropil displayed the lowest activity (dm; Figure 2B). In some glomeruli, the primary olfactory fibers displayed many varicosities and terminal swellings (Figure 3A).

Caudally, in the lateral hemispheres, NADPH-d labeled fibers mostly originated from the evaginated pallium looped dorsomedially to form the stria medullaris (sm; Figure 2C). From here, this bundle of fine fibers then coursed dorsocaudally and can be easily followed toward the habenula (sm; Figures 2D,E, 3D,E), where, at least part of them, crossed the midline in the habenular commissure. More ventrally, numerous thin and weak labeled fibers of unknown origin crossed the midline in the dorsal postoptic commissure (Figures 2D,E).

### Diencephalon

#### Perikarya

Most of the NADPH-d positive diencephalic elements were located in the epithalamus and the pretectum. Both the pineal and parapineal organs presented some labeled cells (Figures 1, 2B,C). However, the most conspicuous epithalamic labeled cell population was located in the right habenula (rHB; Figures 1, 2E, 4A). As it is well known, the lamprey habenula is highly asymmetric, with the right part being much more developed than the left part (see Yáñez and Anadón, 1994; Stephenson-Jones et al., 2012). Most, if not all, cells of the right part were stained for NADPH-d in our experiments, but not all the somata profiles could be readily distinguished because of their small size (around 5 μm in diameter) and their high overlapping (Figure 4A).

In the basal plate of both the anterior and posterior parencephala (p2 and p3), a group of scattered medium-sized (10–15 μm) cells displayed NADPH-d activity. These cells were mainly round and bipolar, and most of them were located close to the ventricular surface (Figures 2G, 4D).

In the caudal diencephalon (p1) of the adult lamprey, numerous neurons were labeled for NADPH-d. Their distinct localization allowed us to distinguish five independent cell groups, three in the alar plate (pretectal region) and two in the basal plate. In the dorsal part of the pretectum, intense NADPH-d activity was present in the subcommissural organ (Figures 1, 2F,G, 4B). This organ is an ependymal gland located bilaterally under the posterior commissure.

Ventrolateral to the subcommissural organ, some NADPH-d positive cells were located dorsomedially to the optic tract in a narrow band (comma-like shape) from the subventricular to almost the subpial surfaces, close to the insertion of the choroid plexus that covers part of the diencephalic roof (Figures 1, 2F,G, 4B). This cell population extended throughout the rostrocaudal length of the pretectum and was labeled as dorsal pretectal nucleus in the drawings of Figures 1, 2. Its labeled cells exhibited long and thin dorsolateral dendrites that ramified in the lateral part of the nucleus (Figure 4B).

In the medial part of the pretectum, there were some NADPH-d labeled cells grouped close to the ventricular surface (medial pretectal nucleus; Figures 1, 2G, 4C). These cells had different size and were the most densely labeled cells in the lamprey brain. They mostly had pear-shaped somata and long dendritic processes that arborized lateral and dorsal to the cell bodies (Figures 2G, 4C).

Two cell populations could be distinguished in the ventral part of the synencephalon. One was formed by cells located mainly periventricularly in and around the midline (periventricular nucleus), and the other was constituted by cells displaced in the ventrolateral aspect of the tegmentum (ventral pretectal nucleus; Figures 1, 2G, 4C). The former was made up by medium-sized (10–15 μm), bipolar or multipolar cells located around the sulcus medius inferior, with some cells migrated ventrally (Figure 4D). Their somata were parallel or perpendicular to the ventricular surface. The main processes of these cells ramified laterally in the ventral or ventrolateral tegmentum, and partially crossed to the contralateral side. In sagittal sections, these cells appeared as a continuation of those located in the basal plate of the two previous prosomeres (p2 and p3; Figure 4D). The second labeled cell population was constituted by smaller (5–8 μm), round-shaped or bipolar cells (Figures 1, 2G, 4C). In transverse sections, this nucleus had a rounded profile with most of the cell processes ramifying locally, inside the nucleus (Figure 4C).

#### Fibers

The thickest fasciculus retroflexus originated from the right part of the habenula shows a moderate NADPH-d activity (Figures 2E–G, 4A,C,D), whereas the thinnest part is almost devoid of labeling. Fibers of this fasciculus coursed directly to the interpeduncular nucleus and constitute the main habenular efferent pathway (see Yáñez and Anadón, 1994). Although these labeled fibers were thin and the intensity of labeling was not high, the course of the fasciculus retroflexus could be easily followed dorsoventrally in the diencephalon (Figures 2E–G, 4A,C,D), to shift caudally in its basal plate and course longitudinally through the mesencephalon and rostralmost part of the rhombencephalon (see below).

### Mesencephalon

#### Perikarya

In the mesencephalon, NADPH-d labeled elements were observed in both the optic tectum and the tegmentum. Within the optic tectum, the largest part of the lamprey mesencephalon, numerous moderate or strong positive cells were located in its mediolateral half part, occupying the stratum cellulare periventriculare and the stratum cellulare et fibrosum internum, as defined by Iwahori et al. (1999; see also de Arriba and Pombal, 2007) (Figures 1, 2H,I, 5A–C). These cells had small (7–10 μm) pear-shaped perikarya with their main dendritic processes perpendicular to the ventricular surface, arborizing mostly in the deep portion of the tectal fiber layers (stratum cellulare et fibrosum externum, stratum opticum and stratum marginale) (Figures 5A–C). Some bipolar cells were also labeled in this region, with a second process running either in the opposite direction or almost parallel to the ventricle. In addition, scattered and weakly labeled round cells of similar size (10 μm in diameter) were located throughout the stratum cellulare et fibrosum externum and the stratum opticum, with some of them displaced in the stratum marginale (particularly in the caudal part of the optic tectum) (Figures 5A–C). Only the proximal part of their processes could sometimes be distinguished in these cells.

Ventrocaudal to the optic tectum, some NADPH-d labeled cells were observed in the periventricular band of cells constituting the torus semicircularis (TS; Figures 1, 2H,I, 5A). These cells were small (5–7 μm), round or pear-shaped, and exhibited a short process in the thin periventricular neuropil and a larger process directed laterally. Within the area of the retinopetal M5 nucleus of Schöber, some cells were also weakly stained (Figures 1, 2H, 5A). They were small (5–7 μm), elongated bipolar cells that characteristically distributed in the periventricular gray dorsal to the oculomotor nucleus and the giant third Müller cell.

Moreover, in the ventral part of the lamprey mesencephalon, a group of moderate to strong NADPH-d positive cells was located in a periventricular position, with some of them ventrally displaced (tegmental periventricular nucleus; Figures 1, 2H, 5D). These cells appear to be the caudal continuation of the periventricular group described in the diencephalic basal plate (see above). In transverse sections of the caudal mesencephalon, this population formed a continuous band from dorsal to the third Müller cell to the same level in the contralateral side (Figures 2H, 5D). Those located ventral to the sulcus medius were small (7–10 μm in diameter) and round, whereas those located more laterally were elongated bipolar cells and slightly larger (Figure 5D).

#### Fibers

No conspicuous tract of fibers was evident in the lamprey mesencephalon, with the exception of the labeled fibers of the fasciculus retroflexus, which located adjacent to the ventral midline on their course toward the interpeduncular nucleus in the rostral rhombencephalon (Figures 2H, 5D). In addition, some weakly labeled thin axons were also seen in the tectofugal tracts (Figures 2I, 6A) at the level of the isthmic region. These labeled fibers seemed to be originated from the NADPH-d positive cells located in the stratum cellulare periventriculare and the stratum cellulare et fibrosum internum of the optic tectum. These tracts coursed first ventrally, in the lateral margin of the brain, and then caudally. Although we could not follow these fibers to their terminal fields, they could be part of the tectobulbar and tectospinal pathways.

### Rhombencephalon

#### Perikarya

NADPH-d labeled elements were observed in both the isthmic region and the rhombencephalon proper.

##### Isthmic Region

In the rostral part of the isthmus, small (5–8 μm) and round or ovoid-shaped NADPH-d positive cells were widely scattered in the tegmentum (rostral isthmic nucleus; Figures 1, 2I, 5C, 6A). Some of these cells were located close to the fourth ventricle whereas others were displaced laterally or lateroventrally (Figure 6A). This nucleus was located at the same dorsoventral level as the anterior rhombencephalic reticular nucleus, but rostrally to the first isthmic Müller cell.

The highest number of NADPH-d positive cells in the lamprey brain was observed in the dorsocaudal isthmic region, where two distinct cell groups could be distinguished by their topographical location (Figures 1, 2J,K). The most conspicuous cell group was located just caudal to the trochlear motor nucleus, and was formed by a band of labeled cells that extended caudally almost to the entrance level of the trigeminal roots (medial isthmic nucleus; Figures 1, 2J, 5C, 6B,C). Its cells were of medium size (10–15 μm), and mainly bipolar with their main axis parallel to the external (pial) surface of the brain. They had numerous dendrites arborizing in different directions, with some reaching and ramifying between the trigeminal sensory fibers and other crossing in the so-called cerebellar commissure (Figures 6B,C). The other cell group was confined in the pretrigeminal region of the caudal isthmic region, just close to the ventricular surface at the level of the sulcus limitans of His (caudal isthmic nucleus; Figures 1, 2K, 6D). The labeled cells were of similar size than those of the previous group, but their dendrites were mostly directed to the alar plate (Figure 6D).

In the ventral isthmic tegmentum, numerous scattered NADPH-d positive cells were observed at both sides of the ventral midline, reaching caudally the level of the trigeminal motor nucleus (Figures 1, 2J–L, 7A). These cells were round or bipolar and small (8–10 μm) and located dorsally to the terminal field of the fasciculus retroflexus, which is also labeled (Figure 7A). In some cases, short processes of these cells could be locally distinguished. Due to the site of distribution, they were identified as belonging to the interpeduncular nucleus (IP); alternatively, these cells could belong to the raphe nucleus (see Section “Discussion”). A few weakly NADPH-d labeled cells were also present in the anterior rhombencephalic reticular nucleus (Figure 1).

##### Rhombencephalon Proper

The medullary sensory dorsal cells located caudally to the entrance of the trigeminal nerve in the rhombencephalic alar plate were also NADPH-d positive (Figures 1, 2M,O, 7B). These cells were large (45–60 μm) and elongated or pear-shaped, but their processes could not be followed from the perikarya. They were located mostly in the subependymal layer along the medial border of the alar plate and were clearly distinguished from the surrounding cells (Figure 7B).

Some cells of the reticular formation were also labeled for NADPH-d. Most of these cells were of medium size (10–15 μm), but some larger cells were also stained (Figures 1, 2J,L,N,O). Curiously, none of the well identified giant reticulospinal cells of the lamprey brain (i.e., M1–M3, I1 and I2, and R1–R8 pairs of Müller cells, as well as the conspicuous Mauthner cells) were labeled in our material (see Figure 5D).

Some small (8–10 μm) NADPH-d positive cells were observed at the level of the sulcus limitans of His in the caudal rhombencephalon (Figure 1). These cells were located close to the ventricular surface but no cell processes could be distinguished. Due to their site of distribution, they were tentatively identified as a subpopulation of the solitary tract nucleus (see Section “Discussion”).

Of interest was the presence of a certain number of NADPH-d labeled cells in the different motor nuclei of the lamprey brain. Only a small proportion of the cells located in the trigeminal (V), facial (VII), glossopharyngeal (IX), vagal (Xr + Xc), and accessory-hypoglossal complex (XI–XII) motor nuclei were weakly to moderate labeled for NADPH-d (Figures 1, 2K–P; see also Figure 7B); however, some cells of the trigeminal (V) motor nucleus were clearly positive (Figure 6D). These cells showed the characteristic dendritic arborization previously described by using retrograde neuronal tracers (Koyama et al., 1987; see Pombal and Megías, 2019), with short apical dendrites ramifying in the periventricular neuropil and larger dendrites ramifying in the ventrolateral tegmentum (see Figure 6D). Some small (8–10 μm), elongated or pear-shaped CSF-c cells were also stained in the ventrolateral part of the caudalmost rhombencephalon (Figures 1, 2P).

#### Fibers

In the dorsal isthmic region, some NADPH-d labeled fibers were observed in the so-called cerebellar commissure. These fibers could partially correspond to the axons of labeled cells belonging to the medial isthmic nucleus. In addition, a compact tract of moderately NADPH-d positive thin fibers ran dorsoventrally in the lateral aspect of the isthmic region. These fibers could be originated from the mesencephalic optic tectum and appeared to course caudally to the basal rhombencephalon.

The labeled fibers coursing in the fasciculus retroflexus (see above) terminated inside the interpeduncular nucleus in the rostral part of the rhombencephalon, including the isthmic region (Figures 2H–L). This nucleus is located in the ventromedial part of the rhombencephalic tegmentum and presented an intense NADPH-d labeled neuropil (see Figures 5D, 7A). In the rostral part of the interpeduncular neuropil, the labeled fibers distributed dorsally, whereas in the caudal part they distributed ventrally (see Figures 2H–L, 5D, 7A).

The strongest stained tract in the rhombencephalon corresponded to the ascending fibers from the dorsal column of the spinal cord (Figures 2J–P). These fibers cover a wide portion of the dorsolateral alar plate in the caudal rhombencephalon, and further split into different small fiber bundles that partially reach the cerebellar plate (see Ronan and Northcutt, 1990; Dubuc et al., 1993). One of these fiber bundles coursed rostralward in the medial octavolateral nucleus, with most fibers located between the electroreceptor and the mechanoreceptor portions of the anterior lateral line nerves that ultimately reach the cerebellar plate (see Figure 7C). The fibers of this bundle characteristically showed bifurcations at the level of the anterior lateral line and octaval nerves as well as large axonal swellings (see Figure 7C).

In sagittal sections, some very weak NADPH-d staining was also present in the descending trigeminal tract (Figures 2K–P); thus, at least part of the sensory fibers coursing in this tract appeared to be positive for NADPH-d.

### Spinal Cord

#### Perikarya

In this study, only the rostralmost part of the spinal cord was analyzed. NADPH-d reaction was present in many structures in the lamprey spinal cord. As it is not possible to recognize a laminar organization of the gray matter, the distribution of NADPH-d positive elements will be framed according to the subdivisions reported in Pombal et al. (1997).

The highest NADPH-d activity was observed in the large sensory dorsal cells (which have similar size to those found in the rhombencephalon). These cells were located dorsolateral to the central canal and ventral to the dorsal horns (Figures 1, 2Q,R, 7D), forming two distinct longitudinal rows on either side of the midline. These cells usually had two coarse processes that originated from their rostral and caudal apices and ran in the dorsal funiculus. They also had one or more additional processes that left the spinal cord via one or more dorsal roots, thus representing first-order mechanosensory neurons (see Nieuwenhuys and Nicholson, 1998). A large number of CSF-c cells also exhibited a moderate to strong NADPH-d activity (Figures 1, 2Q,R, 7D). These cells were small (8–10 μm) elongated or pear-shaped neurons located mostly dorsolateral to the central canal (Figure 7E) and formed a continuous band all along the spinal cord that continued in the caudalmost part of the rhombencephalon (Figure 2P). More dorsally, close to the level of the dorsal cells, other population of cells with the same size exhibited NADPH-d activity (Figures 2Q,R, 7E). Apparently, these cells were not CSF-c cells and formed small and discontinuous groups close to the midline. Only the origin of a lateral directed process could sometimes be distinguished in these cells.

Dorsal to the spinal motor column and ventral to the dorsal horns, a few NADPH-d-exhibiting neurons were located on each side of the cord (Figures 2Q,R, 7D). These cells were small (10 μm), monopolar or bipolar, and had a process oriented toward the lateral column, where it seemed to ramify. Finally, as stated for the motor nuclei of the brainstem, occasionally some neurons of the spinal motor column displayed a weak NADPH-d activity (Figure 7D).

#### Fibers

The dorsal column of the spinal cord showed a relatively intense NADPH-d activity. Numerous labeled fibers could be observed in both the dorsal horns and the dorsomedial columns (Figures 2P–R, 7D). Other NADPH-d labeled scattered fibers distributed throughout the spinal cord, but particularly into and around the cell region.

## Discussion

The present study reports a detailed map of the topographical distribution of NADPH-d positive cells and fibers in the brain and rostral spinal cord of the adult river lamprey, *L. fluviatilis*. The NADPH-d labeled cell populations showed different staining intensities that were included in three major categories: weakly, moderately and strongly labeled. All these cells were undoubtedly identified as neurons, and no glial elements were labeled, although non-neuronal structures such as sunfish tanycytes (Ma, 1993), rainbow trout oligodendrocytes (Pérez et al., 1996), and goldfish glial and endothelial cells (Villani and Guarnieri, 1995b) have been reported to be NADPH-d positive.

### Comparison With Previous Studies in Lampreys

Schober et al. (1994a) reported the distribution of NADPH-d in the CNS of the larval lamprey (*L. planeri*) by using animals between 10 and 14 cm of body length. Due to their body size, these animals could be considered as premetamorphic, i.e., larval lampreys at a late developmental stage. Our results using adult (prespawning) river lampreys (*L. fluviatilis*) presented some similarities with the description made by these authors, but also differed significantly in several aspects.

These differences could be due to the use of different lamprey species (*L. planeri* versus *L. fluviatilis*) and/or different developmental stages (larval versus adult specimens). However, we cannot rule out the possibility of discrepancies due to the fixation procedure. This is a critical step for the histochemical staining of NADPH-d, and excessively short or prolonged fixation may result in a loss of weakly stained elements (see Alonso et al., 2000). Our material was properly fixed during 2–4 h, whereas that of Schober et al. (1994a) was excessively fixed during 24 h.

The most remarkable difference is the low number of positive NADPH-d cells found in larvae of *L. planeri* (Schober et al., 1994a). This is particularly relevant knowing that the presence of NADPH-d activity in the giant Müller and Mauthner cells of the lamprey brainstem reported by these authors, could not be confirmed in the adult specimens used in our experiments. Apart from these giant cells, Schober et al. (1994a) reported NADPH-d activity in the pineal and parapineal organs, the subcommissural organ, and some reticulospinal cells, as well as some CSF-c cells. However, although not explicitly mentioned by the authors, in some of their figures there are other structures that could apparently be also interpreted as NADPH-d positive, such as: mitral cells (see their Figure 5A), periventricular cells in the hypothalamus (see their Figure 5B), cells located dorsally to the M4 pair of Müller cells (see their Figures 3C, 6B), medullary sensory dorsal cells (see their Figures 4A,C, 8A,C), motoneurons of the oculomotor (see their Figure 3B), trigeminal (see their Figures 3D,E), facial (see their Figure 3F), glossopharyngeal (see their Figures 4A, 8A), vagal (see their Figure 4B–D), and accessory-hypoglossal (see their Figures 4E, 8B) motor nuclei, as well as spinal cord motoneurons (see their Figures 4F, 8D). Several spinal cells located dorsal to the central canal as well as some CSF-c cells also seem to be NADPH-d positive (see their Figures 4F, 8D).

In the prosencephalon, the similarities included the intense NADPH-d labeling obtained in the olfactory fibers and glomeruli of the olfactory bulbs, the pineal and parapineal organs, the subcommissural organ, and the habenular region, as well as the lack of staining observed in the putative central pathway of the nervus terminalis (see below; Schober et al., 1994a; present results). The olfactory fibers and glomeruli of the olfactory bulbs were clearly positive for NADPH-d in adult lampreys, where they showed a strong labeling with different intensity. This is in agreement with previous results in larval lampreys of either *L. planeri* (Schober et al., 1994a) or *P. marinus* (Zielinski et al., 1996). In addition, the latter authors reported the presence of NADPH-d activity in olfactory receptor cells, secretory vesicles of sustentacular cells, and basal cells (presumptive progenitors of olfactory receptor cells) of the olfactory mucosa. Based on these results they suggested that nitric oxide could modulate peri-receptor events of L-arginine chemostimulation, olfactory receptor cell axonal activity, and olfactory receptor cell development in the larval lamprey olfactory epithelium (Zielinski et al., 1996).

Our results also show the presence of NADPH-d activity in other regions of the olfactory pathway, including mitral-like cells in the olfactory bulbs and cells in the evaginated pallium projecting via the stria medullaris and the habenula (see also Yáñez and Anadón, 1994; Stephenson-Jones et al., 2012). Some secondary and tertiary olfactory fibers originated from the olfactory bulbs and the evaginated pallium are known to course dorsomedially and caudally, entering the habenular commissure and projecting to several contralateral prosencephalic areas (Northcutt and Puzdrowski, 1988; Polenova and Vesselkin, 1993; Northcutt and Wicht, 1997).

Some NADPH-d activity was detected in both pineal and parapineal organs of either larval (Schober et al., 1994a) and adult (present results) lampreys. A massive staining was also present in the subcommissural organ of larval and adult lampreys (Schober et al., 1994a; present results).

The presence of NADPH-d labeling in the habenula was suggested to be due to the presence of positive pinealofugal cells projecting through the pineal nerve (Schober et al., 1994a). However, only the parapineal organ projects toward the left (smaller) side of the habenula (see Yáñez et al., 1999). In addition, our results demonstrated the presence of numerous small positive cells in the habenula as well as in their axons forming the fasciculus retroflexus. As in other vertebrate species, this fasciculus represents the major efferent tract of the habenula and courses toward the interpeduncular nucleus of the rostral rhombencephalon (Yáñez and Anadón, 1994; Stephenson-Jones et al., 2012). In our experimental material, the NADPH-d positive fibers could be easily followed to the interpeduncular neuropil all along the nucleus.

In relation to the labeled fibers, although Schober et al. (1994a) did not report any staining in either the spinal sensory dorsal cells or the spinal ganglia, most of the dorsal funiculus fibers were labeled in their material (see their Figures 4D–F). Our results corroborated this staining (see below); however, we could not clearly identify labeled fibers in either the anterior lateral line or the octaval nerves, as reported by these authors (see their Figures 3D–F, 4A).

### Comparison With Previous Studies in Other Vertebrates

#### Secondary Prosencephalon

As reported for other vertebrates, different regions of the lamprey olfactory pathway display moderate to strong NADPH-d positive processes and neurons, including the primary olfactory fibers, the evaginated pallium, the stria medullaris and the habenula (Alonso et al., 2000). A strong reaction for NADPH-d was found in the primary olfactory fibers of tetrapods and fish species (Schober et al., 1993; Arévalo et al., 1995; González et al., 1996; Porteros et al., 1996; Singru et al., 2003; Northcutt, 2009; Ferrando et al., 2012; López et al., 2016, 2017, 2019); however, they were shown to be NOS immunonegative in cladistians, holosteans, teleosts, lungfishes, amphibians, and mammals (González et al., 1996; Porteros et al., 1996; Virgili et al., 2001; López and González, 2002; Moreno et al., 2002; Giraldez-Perez et al., 2008, 2013; López et al., 2016, 2017, 2019) due to the presence of cytochrome P-450 reductase, which has close homology with NOS (Bredt et al., 1991b) and presents NADPH-d activity. In fact, the primary afferent olfactory fibers represent the most common example of the variability of the NADPH-d/NOS distribution in the vertebrate CNS. However, in lamprey they were demonstrated to be positive for both NOS (Zielinski et al., 1996) and NADPH-d (present results). These results indicate that NO may play a role in the processing of olfactory inputs, as previously suggested by Breer and Shepherd (1993), Zielinski et al. (1996), and Singru et al. (2003); and the differences among vertebrates could correlate to the developmental degree and importance of the olfactory system of different species (Alonso et al., 2000). The olfactory bulbs of *L. fluviatilis* contain a numerous population of NADPH-d labeled neurons identified like mitral cells (present results). The presence of nitrergic cells in the olfactory bulbs is a variable feature that has also been reported in a teleost fish (Singru et al., 2003), a cartilaginous fish (Ferrando et al., 2012), lungfishes (Northcutt, 2009; López et al., 2019), urodele and gymnophionan amphibians (González et al., 1996, 2002b), and all species of mammals studied (Kishimoto et al., 1993; Hopkins et al., 1996; Kendrick et al., 1997; Alonso et al., 1998). However, nitrergic cells were not detected in the olfactory bulbs of most actinopterygian fishes, anuran amphibians, reptiles and birds.

The presence of a terminal nerve has been described, by using different methods, in members of each class of gnathostomes, where its presence appears to be a primitive condition (see Eisthen and Northcutt, 1996). This nerve showed NADPH-d activity in some groups of fishes and amphibians (Schober et al., 1994b; Brüning and Mayer, 1996; González et al., 1996; Muñoz et al., 1996; López and González, 2002; Singru et al., 2003; López et al., 2016, 2017). Although some authors (Meyer et al., 1987; von Bartheld et al., 1987; Northcutt and Puzdrowski, 1988; von Bartheld and Meyer, 1988) have initially described the projections of a putative terminal nerve in lampreys, these projections may correspond to an extra-bulbar olfactory pathway (Eisthen and Northcutt, 1996; Pinelli et al., 2004; Gayoso et al., 2011). The putative absence of a terminal nerve in lampreys is also chemically supported by the lack of immunoreactivity for both gonadotropin-releasing hormone and FMRFamide (Eisthen and Northcutt, 1996), as well as of NADPH-d activity in both larval and adult lampreys (Schober et al., 1994a; present results).

The evaginated pallium of the evaginated telencephalon of *L. fluviatilis* showed a population of scattered NADPH-d positive cells. Axons of these cells coursed first medially and then dorsocaudally in the stria medullaris, which constitutes a longitudinal tract that crosses to the contralateral side in the habenular commissure. The evaginated pallium receives massive secondary olfactory projections, which in turn projects to different contralateral prosencephalic areas (Polenova and Vesselkin, 1993; Northcutt and Wicht, 1997). The evaginated pallium was also labeled in an equivalent pallial area of cladistians (López et al., 2016), lungfishes (González and Northcutt, 2009; Northcutt, 2009; López et al., 2019), some amphibians (Muñoz et al., 1996) and reptiles (Regidor and Poch, 1988), but not in the equivalent areas of teleosts and holosteans (Arévalo et al., 1995; Gaikwad et al., 2009; López et al., 2017).

The lamprey prethalamic eminence (Pombal et al., 2009) was almost devoid of labeling and the stained cells found along its rostrocaudal extent were identified as belonging to the nucleus of the stria medullaris (Pombal and Puelles, 1999). They were spread all along the stria medullaris, and their axons incorporated to this tract. A portion of our prethalamic eminence is considered by other authors as the lamprey medial pallium (see Pombal and Puelles, 1999; Pombal et al., 2009). However, the medial pallium of lungfishes (González and Northcutt, 2009; López et al., 2019), amphibians (González et al., 1996, 2002b; Muñoz et al., 1996; López and González, 2002) or its equivalent area in cladistians, holosteans and some teleosts (López et al., 2017) housed nitrergic cells.

Of interest is the presence of numerous stained cells in the lamprey amygdala. Although some authors have suggested that this region could correspond to the dorsal pallium of gnathostome vertebrates (Northcutt and Puzdrowski, 1988; Wicht, 1996; Northcutt and Wicht, 1997; Nieuwenhuys and Nicholson, 1998), we have previously proposed that it could be related to a portion of the amygdaloid complex (Martínez-de-la-Torre et al., 2011). The presence of NADPH-d staining has been very useful to identify amygdaloid nuclei in the lungfish (González and Northcutt, 2009; Northcutt, 2009; González et al., 2010; López et al., 2019) and amphibian brain (González et al., 1996; Muñoz et al., 1996; Marín et al., 1998a,b; González et al., 2002b; Moreno and González, 2003, 2005, 2006, 2007), and is also a feature of the amygdaloid complex of reptiles (Brüning et al., 1994; Pitzer and Wirtshafter, 1997), birds (Brüning, 1993; Panzica et al., 1994), as well as mammals (Mizukawa et al., 1989; Pitkanen and Amaral, 1991; Vincent and Kimura, 1992; McDonald et al., 1993; Matsushita et al., 2001), including their counterparts in the everted telencephalon of actinopterygian fishes (González et al., 2014; López et al., 2016, 2017). Therefore, the presence of NADPH-d labeled cells in this population further supports its correspondence with part of the amniote amygdala.

Some NADPH-d positive cells were seen in the dorsal portion of the lamprey striatum and in the septum. In accordance with this result, the presence of nitrergic cells in the striatum and septum is a general feature reported in all sarcopterygian vertebrates, with the exception of the infrared-sensitive snake, *Trimeresurus flavoviridis* (Jiang and Terashima, 1996), which lacks NADPH-d activity in all telencephalic structures. Similarly, the subpallial areas equivalent to basal ganglia and septal regions in the everted telencephalon of actinopterygian fishes (González et al., 2014) also display nitrergic cells (Brüning et al., 1995; Anken and Rahmann, 1996; Ando et al., 2004; Giraldez-Perez et al., 2008, 2013; López et al., 2016, 2017), with the exception of the teleost *Clarias batrachus* (Gaikwad et al., 2009) that lacks nitrergic cells in the telencephalon.

The lateral preoptic nucleus of *L. fluviatilis* contains a population of NADPH-d labeled cells. Comparatively, the presence of nitrergic cells in the preoptic area is a primitive and conserved feature described in all anamniotes studied (see Table 1) and in birds (Brüning, 1993; Cozzi et al., 1997; Atoji et al., 2001), whereas it is only present in some species of reptiles (Smeets et al., 1997) and mammals (Hadeishi and Wood, 1996; Dominguez et al., 2006; Nutsch et al., 2014; Chachlaki et al., 2017; Reis et al., 2018). Functionally, NO in the preoptic region has been related with the control of sexual behavior (Nutsch et al., 2014).

**TABLE 1:** Summary about the distribution of NADPH-d positive/NOS immunoreactive cells in different areas of the central nervous system of the vertebrate groups studied.

	Agnathans	Chondrichthyes	Cladistians	Chondrosteans	Gars	Teleosts	Lungfishes	Amphibians	Reptiles	Birds	Mammals
Olfactory bulb	+	+	−	?	−	±	+	±	−	−	+
Pallium/cortex	+	?	+	?	+	±	+	+	±	+	+
Striatum	+	?	+	?	+	±	+	+	±	+	+
Septum	+	?	+	?	+	±	+	+	±	+	±
Pallidum	1	?	+	?	+	±	+	+	±	+	±
Amygdaloid complex	+	?	+	?	+	±	+	+	±	+	+
Preoptic area	+	?	+	?	+	+	+	+	±	+	±
Paraventricular area	+	+	+	?	+	+	−	±	+	+	+
Suprachiasmatic nucleus	−	?	+	?	+	±	+	±	±	+	±
Basal hypothalamus	+	?	+	+	+	+	+	+	±	+	+
Hypophysis	−	?	−	?	−	±	−	−	?	?	±
Prethalamus	+	?	+	−	+	±	+	±	−	−	±
Habenula	+	?	+	?	+	±	−	±	−	±	−
Thalamus	−	?	+	+	+	±	+	±	±	±	±
Pretectum	+	?	−	?	+	±	+	±	±	+	±
Posterior tubercle VTA/substantia nigra	−	?	+	?	+	±	+	±	±	+	±
Optic tectum/superior colliculus	+	?	+	+	+	+	+	+	+	+	+
Torus semicircularis/Inferior colliculus	+	?	+	?	+	+	+	+	±	±	±
Mesencephalic tegmentum	+	?	+	?	+	+	+	+	+	+	+
Interpeduncular nucleus	+	?	+	?	−	±	+	±	±	+	±
Cerebellum	−	?	−	+	−	+	−	±	±	±	+
Cerebellar nucleus	−	+	+	?	+	?	+	±	±	±	±
Non-motor nuclei in the upper rhombencephalon: LDT/PPT/Rs	+	?	−	?	−	±	+	+	+	+	+
Locus coeruleus	−	?	−	?	−	±	−	−	−	±	−
Reticular formation	+	?	+	+	+	+	+	+	+	+	+
Octavolateral area	−	?	+	?	+	+	+	±	±	+	+
Raphe nuclei	2	?	−	?	−	±	−	±	±	±	±
Motor nuclei of the cranial nerves	3	?	−	?	−	±	−	±	±	±	±
Solitary tract nucleus	+	?	+	?	+	±	+	±	±	+	±
Spinal cord	+	?	+	?	+	+	+	+	+	+	+

*(+) Presence of labeled cells; (−) absence of labeled cells; (±) presence of labeled cells in some species of the group; (?) no data available; (1) a putative pallidum was suggested by Stephenson-Jones et al. (2011) to be located in the area interpreted by us as prethalamic eminence (Pombal et al., 2009); therefore, at least part of the cells included in our NSM appear to distributed in the putative pallidum; (2) some of the NADPH-d labeled neurons at both sides of the rostral rhombencephalon could belong to the raphe nuclei (see Section “Discussion”); (3) only some motoneurons showed weakly NADPH-d staining, but some trigeminal neurons were clearly positive. This table has been modified from López et al. (2019; see there the references used for the different species).*

Within the hypothalamus, the caudal paraventricular area of *L. fluviatilis* housed a group of small NADPH-d labeled cells and projections to the hypophysis could not be confirmed (present results); however, the vasotocinergic (Pombal and Puelles, 1999) and cholinergic (Pombal et al., 2001) cells reported in this area project to the hypophysis. Comparatively, nitrergic cells are present in the paraventricular area of teleosts (Schober et al., 1993; Arévalo et al., 1995; Anken and Rahmann, 1996; Gaikwad et al., 2009), cladistians and holosteans (López et al., 2016, 2017), and similar nitrergic groups with hypothalamo-hypophysial projections have been reported in the electric ray (Pérez et al., 1995), and in anuran amphibians (Allaerts et al., 1997; Prasada-Rao et al., 1997), although some amphibian and lungfish species lack nitrergic cells in the paraventricular region (Pinelli et al., 2014; López et al., 2019). In mammals, the NADPH-d positive cells of the neurosecretory hypothalamic nuclei co-express vasopressin/oxytocin (Arévalo et al., 1992; Miyagawa et al., 1994; Sánchez et al., 1994), where the NO probably modulates the release of these neurohormones (Vanhatalo and Soinila, 1995), but this colocalization is absent in the hypothalamus of birds (Sánchez et al., 1996). Thus, whereas the presence of nitrergic cells in the paraventricular region of anamniotes seems to be a primitive and variable feature, in amniotes it is a widely reported feature.

In the ventrocaudal hypothalamus of the river lamprey, NADPH-d labeled cells were found in the hypothalamic periventricular organ. Although there are some problems to unambiguously identify this nucleus in many other species (for example, some authors apparently use for this nucleus the term “paraventricular nucleus/area,” which is now used for a region in the alar hypothalamus), similar results were found only in some species of teleosts (Villani and Guarnieri, 1995a; Pushchina and Obukhov, 2010), chicken (Brüning, 1993; Montagnese and Csillag, 1996), and rats (Vanhatalo and Soinila, 1995).

Within the caudal hypothalamus of *L. fluviatilis* small NADPH-d labeled cells with CSF-c processes were observed in the mamillary region (present results). The presence of nitrergic cells in specific locations of the basal hypothalamus is a shared and highly conserved feature reported in all vertebrate groups (see Table 1), with the only exception described in the snake *T. flavoviridis*, which lacks NADPH-d reactive cells in the whole basal hypothalamus (Jiang and Terashima, 1996).

#### Diencephalon

In the epithalamus of the lamprey, NADPH-d positive elements were observed in both the pineal and parapineal organs, as well as in the highly asymmetric habenula. Several authors have reported the presence of NADPH-d positive cells in the pineal organ of fish (Anken and Rahmann, 1996; Zipfel et al., 1999; Westermann and Meissl, 2008) and frogs (Sato, 1990; Guglielmotti and Fiorino, 1999), as well as in the habenula of different anamniote vertebrates (Villani et al., 1994; Villani and Guarnieri, 1995a,b; Guglielmotti and Fiorino, 1999). However, they were not present in other fish (Arévalo et al., 1995; Brüning et al., 1995), and amphibian species (González et al., 1996).

In the dorsalmost part of alar p2, the asymmetric habenula of *L. fluviatilis* contains a prominent population of NADPH-d labeled cells strongly compacted in the large right habenula whose processes can be observed in the fasciculus retroflexus toward the interpeduncular nucleus. In cladistians, nitrergic cells were only observed in the ventral part of left habenula (López et al., 2016), whereas in holosteans more numerous nitrergic cells were located in the ventrolateral part of the large right habenula and the smaller left habenula, separated from the cholinergic habenular population (López et al., 2017). The presence of nitrergic cells in the habenula is variable in teleosts and amphibians. Thus, nitrergic cells were observed dorsally located in the habenula of the goldfish and almost one third colocalized ChAT (Giraldez-Perez et al., 2008, 2009), whereas in amphibians these cells have been described only in the ventral habenula of *Rana* and *Dermophis* (Muñoz et al., 1996; González et al., 2002b) and nitrergic habenular asymmetry was found only in *Rana esculenta* (Guglielmotti and Fiorino, 1999). Habenular nitrergic cells are absent in lungfishes (López et al., 2019) and almost all amniotes with the exceptions reported only in two avian species (Brüning, 1993; Atoji et al., 2001). The NO in the habenula could be related to the regulation of fear and the reward system (Agetsuma et al., 2010; Jesuthasan, 2012).

Also, in the alar plate of p2 and ventral to the habenula, the thalamus of the river lamprey lacks NADPH-d positive cells. However, the existence of nitrergic cells in the thalamus is a widely observed feature in other anamniotes, like cladistians, chondrosteans, holosteans, teleosts and amphibians (especially remarkable in anurans; see Table 1), with the exceptions of the teleost *C. batrachus* (Gaikwad et al., 2009) and the urodele amphibian *Pleurodeles waltl* (González et al., 1996). In contrast, this feature is rare in amniotes, and it has only been described in the thalamus of the lizard *Gekko gecko* (Smeets et al., 1997), in the Japanese quail and budgerigar (Panzica et al., 1994; Cozzi et al., 1997), and in the rat (Vincent and Kimura, 1992).

Several NADPH-d labeled cell populations are present in the lamprey synencephalon; however, apart from the subcommissural organ, no clear correlation can be made with other cell populations in this region previously identified experimentally. Thereby, we decided to name them as dorsal, medial, ventral, and periventricular pretectal nuclei. The presence of pretectal nitrergic cells is a feature described in holosteans, lungfishes, birds, most species of teleosts and amphibians, and some species of reptiles and mammals (see Table 1), whereas they are absent in cladistian fishes (López et al., 2016). Therefore, the presence of pretectal cells seems a primitive and variable feature in the evolution of the nitrergic system. Due to their location in the dorsal pretectum, their rostrocaudal distribution and their dendritic plexuses, the NADPH-d positive cells of the dorsal pretectal nucleus could correspond to the parapinealopetal cells previously reported by Yáñez et al. (1999).

#### Mesencephalon

In the lamprey optic tectum, the largest part of the NADPH-d labeled cells is found in the stratum cellulare periventriculare and the stratum cellulare et fibrosum internum. Due to their morphology, these cells appear to be projecting cells. The presence of nitrergic cells in periventricular tectal locations is a primitive and conserved feature described in all groups of vertebrates (see Table 1), probably involved in multisensory integration.

A second population of NADPH-d positive cells in the lamprey optic tectum was found in the more superficial stratum cellulare et fibrosum externum and stratum opticum. These cells could correspond to those transneuronally labeled from the eye orbit in the same species of lamprey (de Arriba and Pombal, 2007). Comparatively, nitrergic cells have also been reported in outer tectal layers of chondrosteans, teleosts, reptiles and birds (Brüning, 1993; Meyer et al., 1994; Villani and Guarnieri, 1995a; Cozzi et al., 1997; Smeets et al., 1997; Atoji et al., 2001; Scicolone et al., 2006; Pushchina and Obukhov, 2012). In mammals, nitrergic cells are distributed in inner, intermediate and outer layers of the superior colliculus (Mizukawa et al., 1989; Vincent and Kimura, 1992; Egberongbe et al., 1994), and have been proposed to be interneurons that convey cortical influences onto superior colliculus output neurons (Fuentes-Santamaria et al., 2008).

Ventrocaudal to the optic tectum, small NADPH-d labeled cells were observed periventricularly in the torus semicircularis of river lamprey. The presence of toral nitrergic cells is also a primitive and conserved feature reported in all groups of anamniotes and in most amniotes studied (see Table 1). The exceptions noted, using NADPH-d reaction, reported lack of labeled cells in the torus of the snake *T. flavoviridis* (Jiang and Terashima, 1996), the budgerigar *Melopsittacus undulatus* (Cozzi et al., 1997), and the inferior colliculus of the cat (Mizukawa et al., 1989). The toral nitrergic cells are probably related with the processing of auditory information (Grassi et al., 1995; Wu et al., 2008).

A group of NADPH-d labeled cells is located within the mesencephalic tegmentum of the river lamprey, whereas the oculomotor nucleus lacks nitrergic labeling (present results). All groups of vertebrates studied show a characteristic nitrergic cell population in the mesencephalic tegmentum representing another primitive and conserved feature (see Table 1). A shared feature of amniotes is the presence of the dopaminergic ventral tegmental area/substantia nigra (VTA/SN) complex in the midbrain tegmentum (Smeets and González, 2000). By means of double labeling techniques many of these dopaminergic cells have been demonstrated to co-express NOS in lungfishes, reptiles, birds and mammals (Johnson and Ma, 1993; Panzica et al., 1996; Smeets et al., 1997; López et al., 2019). However, the counterpart of the VTA/SN complex in lampreys most likely lies in the diencephalic posterior tubercle (Pombal et al., 1997; Ericsson et al., 2013), where no nitrergic cells were observed (present results).

#### Rhombencephalon

Three NADPH-d labeled cell populations were identified in the dorsal isthmic region of the lamprey brain. According to their relative topographical position, they were named as rostral, medial and caudal isthmic nuclei. The rostral isthmic nucleus is located in the most rostral part of the isthmic region, just delineating the limit between the isthmus and the rostrally located mesencephalon.

The medial isthmic nucleus is located caudally and ventrally to the trochlear motor nucleus. Cells of this nucleus could belong to the lamprey laterodorsal tegmental nucleus (LDT) characterized by the presence of cholinergic cells in other vertebrates. The presence of cholinergic cells in this region was previously reported (Pombal et al., 2001). The LDT and its related pedunculopontine tegmental nucleus (PPN) co-express both acetylcholine and NOS in lungfishes (López et al., 2019), amphibians (González et al., 1996, 2002a; Muñoz et al., 1996, 2000; López and González, 2002) and amniotes (Alonso et al., 2000; Veleanu et al., 2016). This cholinergic and nitrergic complex is related with important functions like the control of sleep-wake cycle, learning, reward system and motor control (Wang and Morales, 2009). Of note, double labeling experiments in cladistian and holostean fishes do not show nitrergic labeling in the cholinergic cells of this nucleus (López et al., 2016, 2017). The absence of colocalization of NOS and acetylcholine in the LDT found in these basal actinopterygian fishes could represent a primitive neurochemical feature of this important cholinergic group that must be analyzed in the future in the lamprey brain to test this hypothesis.

At pretrigeminal level, the NADPH-d labeled neurons identified as caudal isthmic nucleus located at the level of the sulcus limitans of His, between the trigeminal motor nucleus and the octavolateral area. This is the region occupied by the lamprey visceral sensory zone, with the ascending fibers of the solitary tract reaching the rostral rhombencephalon (Pombal and Megías, 2019).

Some NADPH-d positive cells were found at both sides of the ventral midline in the rostral part of the lamprey rhombencephalon. Due to their position, two possibilities were considered to identify this cell population. First, these cells could correspond to the lamprey interpeduncular nucleus, because they located just over the dense interpeduncular neuropil formed by the terminal fibers of the fasciculus retroflexus. The presence of nitrergic cells in this nucleus is an uncommon feature in anamniotes, particularly described in cladistians (López et al., 2016), the teleost *Carassius auratus* (Giraldez-Perez et al., 2008), the lungfish *Neoceratodus forsteri* (López et al., 2019), and the anuran amphibian *Hyla septentrionalis* (Pinelli et al., 2014). Among amniotes, these cells are present in all species of birds studied and in most mammals, with the exception of humans (Egberongbe et al., 1994), whereas in reptiles they were only described in the turtle *Pseudemys scripta elegans* (Brüning et al., 1994). Second, they could be part of the raphe nucleus in the lamprey. An extensive serotoninergic system is present in the raphe region of gnathostomes and several serotoninergic cell groups were identified in the rostral rhombencephalon of the lamprey (Pierre et al., 1992); however, these authors could not establish precise homologies between these populations in lamprey and those described in gnathostomes. The presence of nitrergic cells in the raphe nuclei has been described in representatives of most vertebrate groups, but using exclusively single labeling techniques (teleosts: Arévalo et al., 1995; Pushchina and Diuizen, 2004; Giraldez-Perez et al., 2008; amphibians: González et al., 1996, 2002b; Muñoz et al., 1996; Lázár and Losonczy, 1999; Huynh and Boyd, 2007; reptiles: Smeets et al., 1997; birds: Atoji et al., 2001; mammals: Vincent and Kimura, 1992; Egberongbe et al., 1994). However, reliable data obtained with double labeling techniques revealed NOS in serotoninergic neurons in the rostral raphe nuclei of the rat (Johnson and Ma, 1993; Dun et al., 1994; Wotherspoon et al., 1994; Wang et al., 1995; Simpson et al., 2003; Lu et al., 2010), but not in the raphe nuclei of mice, guinea pigs and cats (Leonard et al., 1995; Léger et al., 1998). Additionally, previous double labeling studies have demonstrated that only the cells in the caudal part of the raphe column in amphibians also express NOS (López et al., 2005), but total absence of colocalization in the raphe column of cladistians, holosteans and lungfishes (López et al., 2016, 2017, 2019). Therefore, the presence of NOS in the raphe nuclei seems to be another variable feature that requires precise experimental confirmation in many vertebrate groups.

A small population of medium to large and weak NADPH-d labeled cells was observed in the reticular formation of the river lamprey (present results). The presence of nitrergic cells in the reticular formation is one of the most conserved traits of the nitrergic system, described in all vertebrates studied (see Table 1). These cells may probably be reticulospinal neurons, as it has been demonstrated in amphibians (McLean and Sillar, 2000; González and ten Donkelaar, 2007; Sánchez-Camacho et al., 2001).

NADPH-d activity was present in the sensory dorsal cells located either in the alar plate of the medulla, close to the ventricle, or in the spinal cord, dorsolaterally to the central canal. These cells are primary mechanosensory neurons responding to touch, pressure, and nociceptive stimulation of the skin (Rovainen, 1967, 1979; Rovainen and Yan, 1985). Part of these cells, corresponding to those located in the medulla and rostral spinal cord, were named as primary medullary and spinal nucleus of the trigeminal nerve (PMSV), because they are labeled through the trigeminal nerve (Finger and Rovainen, 1982; Anadón et al., 1989). The rest of these cells, all located in the spinal cord, have a peripheral process coursing in the dorsal spinal roots. In addition, PMSV cells were suggested to be homologous of the mesencephalic trigeminal nucleus present in all gnathostome vertebrates (Northcutt, 1979; Anadón et al., 1989). The mesencephalic trigeminal nucleus was also reported to display NADPH-d activity in goldfish (Brüning et al., 1995), swordtail fish (Anken and Rahmann, 1996), and the frog *Xenopus laevis* (Brüning and Mayer, 1996), and was weakly stained in *Pleurodeles waltl* (González et al., 1996), chicken (Brüning, 1993), quail (Panzica et al., 1994), and cat (Lazarov and Dandov, 1998). However, these cells have been shown to be negative for NADPH-d in *Rana* (Muñoz et al., 1996; Lázár and Losonczy, 1999) and *Dermophis* (González et al., 2002b), as well as NOS immunonegative in *Pleurodeles waltl* and basal actinopterygian fishes (González et al., 1996; López et al., 2016, 2017).

Some motoneurons of VII, IX, X, XI–XII and especially of V showed NADPH-d activity in the hindbrain of the river lamprey (present results). Interestingly, motoneurons of the three nuclei innervating the extraocular muscles (oculomotor, trochlear, and abducent nuclei) were negative for NADPH-d. The general weak reaction of the motoneurons (with the exception of V), together with the smaller size of the motoneurons of these three nuclei, could account for this result. The presence of nitrergic staining (generally NADPH-d activity) in the motor nuclei of the cranial nerves is a feature mentioned in some species of teleosts (Arévalo et al., 1995; Villani and Guarnieri, 1995a; Anken and Rahmann, 1996; Pushchina, 2007; Giraldez-Perez et al., 2009), amphibians (Muñoz et al., 1996; Lázár and Losonczy, 1999; Huynh and Boyd, 2007; Pinelli et al., 2014), reptiles (Bennis et al., 1996; Jiang and Terashima, 1996), birds (Brüning, 1993; Atoji et al., 2001), and mammals (Vincent and Kimura, 1992; Varathan et al., 2001; Chertok and Kotsyuba, 2014). However, consistent evidence obtained with double immunolabeling techniques for NOS and ChAT has been positively performed in the case of goldfish (Giraldez-Perez et al., 2009). In contrast, similar double labeling techniques demonstrated absence of nitrergic labeling in the motoneurons of the cranial motor nuclei of cladistians, holosteans and lungfishes (López et al., 2016, 2017, 2019). Therefore, the localization of NOS in rhombencephalic motoneurons is a feature that needs confirmation with the appropriate double labeling techniques.

In the caudal rhombencephalon of *L. fluviatilis*, a NADPH-d positive cell group is observed in the solitary tract nucleus (NTS), which constitutes a discrete subpopulation of this nucleus. Similar data have been reported in cladistians, holosteans and lungfishes (López et al., 2016, 2017, 2019), whereas nitrergic cells in the NTS of teleosts are only described in the tench (Arévalo et al., 1995). In tetrapods, this is a general feature observed in birds and most species of amphibians, with the exception of *H. septentrionalis* (Pinelli et al., 2014), and mammals, with the exception of the cat (Mizukawa et al., 1989). In reptiles, the NTS contains nitrergic cells in the turtle and lizard (Brüning et al., 1994; Smeets et al., 1997), but not in chameleons and snakes (Bennis et al., 1996; Jiang and Terashima, 1996). The NO in cells of NTS has been related with the modulation of visceral sensory afferents, the control of blood pressure, and the regulation of the respiratory frequency (Lawrence et al., 1998; Wu et al., 2002; Granjeiro et al., 2009; Kong et al., 2009).

#### Spinal Cord

In the lamprey spinal cord, white and gray matter can be histologically distinguished, corresponding to regions of nerve fibers and regions of cells respectively, but dorsal and ventral horns are absent (see Nieuwenhuys and Nicholson, 1998). Although the number of NADPH-d labeled cells is not too high, several groups could be distinguished in the lamprey spinal cord. As occurred in the medulla, the sensory dorsal cells of the spinal cord were strongly reactive for NADPH-d. The fibers of the extramedullary and intramedullary primary sensory cells together constitute a distinct fiber bundle, the dorsal funiculus of Nieuwenhuys and Nicholson (1998). Many of these fibers terminate in the caudal rhombencephalon (nucleus funiculi dorsalis of Nieuwenhuys and Nicholson, 1998), but some continue further rostrally to reach the octavolateral area and the cerebellum (Ronan and Northcutt, 1990; Dubuc et al., 1993). In addition, Martin and Bowsher (1977) provided neurophysiological evidence that in the ammocete the axons of many dorsal cells extend as far as the isthmic region. The extramedullary fibers originate from spinal ganglion cells aggregated in true spinal ganglia. In this work, we did not study the spinal ganglia; nevertheless, most spinal ganglion cells of the frog were found to be strongly positive for NADPH-d (Crowe et al., 1995; González et al., 1996; Muñoz et al., 2000), similarly to the situation observed in the sensory ganglion cells of cranial nerve in cladistian fishes (López et al., 2016).

An important feature is the presence of NADPH-d positive CSF-c cell bodies in the caudal rhombencephalon and spinal cord of the river lamprey. So far, the presence of this type of labeled CSF-c cells is a primitive feature that had not been previously reported in any other vertebrate (Alonso et al., 2000); however, in the lamprey it is quite common to find this type of cells immunoreactive for different neurotransmitters/neuromodulators (see below).

Additionally, some other positive cells were found either dorsally to the central canal or laterally in the dorsal part of the gray matter. NADPH-d labeled cells were observed in both the dorsal and ventral horns of diverse mammalian and non-mammalian species. The occurrence of spinal nitrergic cells mainly located in the dorsal horn is an ancient and highly conserved feature of all vertebrates, from lampreys to mammals (see Table 1). In addition, nitrergic cells have been reported in the intermediate and/or ventral spinal gray of actinopterygian fishes (Arévalo et al., 1995; Brüning et al., 1995; Giraldez-Perez et al., 2008; López et al., 2016, 2017), lungfishes (López et al., 2019), amphibians (Crowe et al., 1995; González et al., 1996, 2002b; Muñoz et al., 1996, 2000; Huynh and Boyd, 2007), reptiles (Jiang and Terashima, 1996; Radmilovich et al., 1997; Smeets et al., 1997; Morona et al., 2006, 2007), birds (Atoji et al., 2001; Necker, 2004, 2005), and mammals (Dun et al., 1992, 1993; Terenghi et al., 1993; Vizzard et al., 1994, 1997; Doone et al., 1999; Kluchová et al., 2002; Lukácová et al., 2002; Bombardi et al., 2013). However, there are many differences related to the number, morphology and location between species, and no clear correspondence can be made with those found in the lamprey spinal cord.

Some NADPH-d positive cells were found in the spinal motor column of the river lamprey, which correspond to somatic motoneurons. The presence of nitrergic somatic motoneurons in the spinal cord of vertebrates is an unusual feature that has been reported only in some species of teleosts (Pushchina et al., 2007; Giraldez-Perez et al., 2009) and mammals (Dun et al., 1992; Terenghi et al., 1993; Wetts and Vaughn, 1994) but not in cladistians, holosteans and lungfishes with double labeling techniques (López et al., 2016, 2017, 2019). Although it was not reported, some motoneurons also appear to be positive in the larval lamprey spinal cord (Schober et al., 1994a; see their Figures 4F, 8D). Of note, effect of NO in the regulation of the locomotor activity has been demonstrated in the lamprey spinal cord (Kyriakatos et al., 2009), and other functions for NO in the spinal cord are related with the inhibition of the nociceptive information and pain control (Hervera et al., 2010; Chu et al., 2011; Cury et al., 2011; Jin et al., 2011, 2012).

## Conclusion

This study represents the first detailed neuroanatomical description of the distribution of the NADPH-d labeled (nitrergic) cells and fibers in the central nervous system of the adult lamprey. Apart from the description of the complex nitrergic system, the technique used in our study was confirmed as a powerful tool for the identification of cell groups and brain structures that are otherwise indistinct in the lamprey brain. Distinct patterns of distribution of NADPH-d positive cells and fibers were observed in the forebrain, brainstem and spinal cord. The analysis of the nitrergic system in the brain is very useful in neuroanatomy and, therefore, it has been studied in almost all groups of vertebrates to help in the clarification of neuron types and the organization of particular brain regions. For the interpretation of the distribution patterns of NADPH-d activity in the river lamprey, we have used of the current neuromeric brain model, which has been validated for most vertebrates (forebrain: Puelles and Rubenstein, 2003; Pombal and Puelles, 1999; Pombal et al., 2009; Puelles and Rubenstein, 2015; brainstem: Gilland and Baker, 1993; Aroca and Puelles, 2005; Straka et al., 2006). This approach is optimal for identifying structures that have topologically identical positions, even if their position in adult brains differs across vertebrate species because of specific morphogenetic developments. The current results show a pattern of organization of the nitrergic structures that could represent the ancient pattern of this neurotransmission system. Comparatively with other groups of vertebrates analyzed, several features of the nitrergic system are highly conserved in the nervous system, such as the presence of nitrergic cells in pallial/cortical areas, striatum, amygdaloid complex, basal hypothalamus, mesencephalic optic tectum, torus semicircularis and tegmentum, the rhombencephalic reticular formation, and the spinal cord. This highly preserved pattern of distribution of the nitrergic system suggests that nitric oxide might be involved from early in the evolution of vertebrates in functions such as the motor control, the emotion/behavior control, the regulation of the hypothalamic functions, and the modulation of sensory information in midbrain areas and the spinal cord. On the contrary, the presence of nitrergic cells in the olfactory bulb, pretectum, and interpeduncular nucleus, or its colocalization with cholinergic motoneurons of cranial nerves/spinal cord and the serotonergic cells in the raphe nuclei, are variable features. Furthermore, peculiar characteristics of the nitrergic system of lamprey include the absence of nitrergic labeling in the posterior tubercle and the numerous nitrergic CSF-c cells in the caudal rhombencephalon and spinal cord.

## Data Availability Statement

The original contributions presented in the study are included in the article, further inquiries can be directed to the corresponding author.

## Ethics Statement

The animal study was reviewed and approved by Xunta de Galicia, Consellería do Medio Rural Xefatura Territorial de Pontevedra, Servizo de Gandaría Avda. María Victoria Moreno, 43, 2° 30071-Pontevedra, Spain.

## Author Contributions

MAP and JML: study concept and design, analysis and interpretation of data, and drafting of the manuscript. MM, DL, and JML: acquisition of data. MAP and MM: figure preparation. All authors corrected, edited, and approved the manuscript.

## Conflict of Interest

The authors declare that the research was conducted in the absence of any commercial or financial relationships that could be construed as a potential conflict of interest.

## Publisher’s Note

All claims expressed in this article are solely those of the authors and do not necessarily represent those of their affiliated organizations, or those of the publisher, the editors and the reviewers. Any product that may be evaluated in this article, or claim that may be made by its manufacturer, is not guaranteed or endorsed by the publisher.

## Footnotes

1
https://www.gimp.org/

2
https://inkscape.org/

## Abbreviations

A: amygdala
AB: anterobasal nucleus
AH: anterior hypothalamus
aot: anterior octavomotor tract
arn: anterior rhombencephalic reticular nucleus
cc: central canal of the spinal cord
ch: optic chiasma
cI: caudal isthmic nucleus
CPa: caudal paraventricular area
cp: cerebellar plate
cpt: posttectalis commissure
cs: central stratum (optic tectum)
CSF: CSF-c neurons of the spinal cord
CSF-c: cerebrospinal fluid-contacting cells
D: diencephalon
dc: dorsal cells
dcf: dorsal column fibers
dh: dorsal horn of the spinal cord
dm: dorsomedial telencephalic neuropil
dmc: dorsomedial column of the spinal cord
dn: dorsal nucleus of the octavolateral area
dpoc: dorsal postoptic commissure
dPT: dorsal pretectal nucleus
ds: dorsal sac
dV: descending trigeminal tract
eALL: electrosensory lateral line
EP: evaginated pallium
EPd: dorsal part of the evaginated pallium
EPv: ventral part of the evaginated pallium
f: fasciculus retroflexus
go: glomeruli olfactorii
HB: habenula
hp1: hypothalamic prosomere 1 (peduncular or caudal hypothalamus)
hp2: hypothalamic prosomere 2 (prepeduncular or rostral hypothalamus)
igl: internal granular layer of the olfactory bulb
III: oculomotor nucleus
IP: interpeduncular nucleus
IV: trochlear motor nucleus
IX: glossopharyngeal motor nucleus
lc: lateral column of the spinal cord
lHB: left habenula
LPO: lateral preoptic nucleus
ltr: lateral tectal recess
M: mesencephalon
M3: third Müller cell (mesencephalic)
M5: nucleus M5 of Schöber
ma: axons of reticulospinal cells
mALL: mechanosensory lateral line
MAM: mamillary region *sensu lato*
mI: medial isthmic nucleus
mlf: medial longitudinal fasciculus
mn: medial nucleus of the octavolateral area
MPO: medial preoptic nucleus
mPT: medial pretectal nucleus
mrn: medial rhombencephalic reticular nucleus
mt: mesencephalic tegmentum
NH: neurohypophysis
nIII: oculomotor nerve
NSM: nucleus of the stria medullaris
nTPOC: nucleus of the tract of the postoptic commissure
NTS: nucleus of the solitary tract
nV: trigeminal nerve
nVI: abducens nerve
OB: olfactory bulb
on: optic nerve
os: optic stratum (optic tectum)
OT: optic tectum
P: pineal organ
p1–p3: prosomeres 1–3
pc: posterior commissure
pch: choroidal plexus
pD: periventricular diencephalic neurons
PE: prethalamic eminence
PEP: entopeduncular nucleus
pM: periventricular mesencephalic neurons
PP: parapineal organ
pPT: periventricular pretectal neurons
prn: posterior rhombencephalic reticular nucleus
Ps: pineal stalk
ps: periventricular stratum (optic tectum)
PT: pretectum
pto: periventricular cells of the optic tectum
PVO: hypothalamic periventricular organ
R: rhombencephalon
rh1–rh8: rhombomeres 1–8
rHB: right habenula
rI: rostral isthmic nucleus
ri: infundibular recess
RM: retromamillary area
RPa: rostral paraventricular area
rpo: postoptic recess
rpro: preoptic recess
S: striatum
SC: spinal cord
SCO: subcommissural organ
SE: septum
sm: stria medullaris
smc: spinal motor column
SPr: secondary prosencephalon
ss: superficial stratum (optic tectum)
sto: superficial cells of the optic tectum
Th: thalamus
TM: tuberomamillary nucleus
TN: tuberal nucleus
to: optic tract
TS: torus semicircularis
V: trigeminal motor nucleus
vc: ventral column of the spinal cord
vd: diencephalic ventricle
VI: abducens motor nucleus
VII: facial motor nucleus
vla: anterior lateral telencephalic ventricle
vlp: posterior lateral telencephalic ventricle
vm: mesencephalic ventricle
vn: ventral nucleus of the octavolateral area
vPT: ventrolateral pretectal nucleus
vrh: rhombencephalic ventricle
vt: impar telencephalic ventricle
X: vagal motor nucleus
Xc: vagal motor nucleus (caudal part)
XI: accessory motor nucleus
XII: hypoglossal motor nucleus
Xr: vagal motor nucleus (rostral part).
